# BAG3 as a novel prognostic biomarker in kidney renal clear cell carcinoma correlating with immune infiltrates

**DOI:** 10.1186/s40001-024-01687-w

**Published:** 2024-02-01

**Authors:** Binghao Gong, Yuan Huang, Zhenting Wang, Bangbei Wan, Yaohui Zeng, Cai Lv

**Affiliations:** 1 Department of Urology, Central South University Xiangya School of Medicine Affiliated Haikou Hospital, Haikou, China; 2 Department of Neurology, Central South University Xiangya School of Medicine Affiliated Haikou Hospital, Haikou, China

**Keywords:** BAG3, Prognosis, Biomarker, Immune infiltrate, Kidney clear cell carcinoma

## Abstract

**Purpose:**

BCL-2-associated athanogene 3 (BAG3) is an anti-apoptotic protein that plays an essential role in the onset and progression of multiple cancer types. However, the clinical significance of BAG3 in kidney renal clear cell carcinoma (KIRC) remains unclear.

**Methods:**

Using Tumor IMmune Estimation Resource (TIMER), The Cancer Genome Atlas (TCGA), and Gene Expression Omnibus (GEO) database, we explored the expression, prognostic value, and clinical correlations of BAG3 in KIRC. In addition, immunohistochemistry (IHC) of HKH cohort further validated the expression of BAG3 in KIRC and its impact on prognosis. Gene Set Cancer Analysis (GSCA) was utilized to scrutinize the prognostic value of BAG3 methylation. Gene Ontology (GO) term analysis, Kyoto Encyclopedia of Genes and Genomes (KEGG), and Gene set enrichment analysis (GSEA) were used to identify potential biological functions of BAG3 in KIRC. Single-sample gene set enrichment analysis (ssGSEA) was performed to confirm the correlation between BAG3 expression and immune cell infiltration.

**Results:**

BAG3 mRNA expression and protein expression were significantly downregulated in KIRC tissues compared to normal kidney tissues, associated with adverse clinical–pathological factors and poor clinical prognosis. Multivariate Cox regression analysis indicated that low expression of BAG3 was an independent prognostic factor in KIRC patients. GSEA analysis showed that BAG3 is mainly involved in DNA methylation and the immune-related pathways in KIRC. In addition, the expression of BAG3 is closely related to immune cell infiltration and immune cell marker set.

**Conclusion:**

BAG3 might be a potential therapeutic target and valuable prognostic biomarker of KIRC and is closely related to immune cell infiltration.

**Supplementary Information:**

The online version contains supplementary material available at 10.1186/s40001-024-01687-w.

## Introduction

Renal cell carcinoma (RCC) is one of the most common malignancies in the urinary system, with approximately 431,000 new cases and 179,000 new deaths globally in 2020 [1]. Renal cell carcinoma contains several histological subtypes, among which KIRC is the most common, accounting for about 75% of cases [2], with smoking, obesity, and hypertension being the most significant risk factors [3]. Surgery remains the primary therapeutic option for localized RCC [4]. However, patients with metastatic ccRCC have a terrible prognosis, with a 5-year survival rate of fewer than 10% [5]. With the rapid development of targeted agents and immune checkpoint inhibitors (ICIs) in recent years, the 5-year survival rate of patients with advanced KIRC has improved, but the prognosis is still unfavorable overall [6, 7]. In addition, the widespread use of targeted drugs and immunologic agents inevitably triggers drug resistance and adverse effects [8]. For instance, patients receiving treatment with ICI and anti-vascular endothelial growth factor (VEGF) exhibited a markedly elevated risk of clotting disorders connected to the heart and blood [9]. Therefore, an in-depth exploration of novel diagnostic biomarkers of KIRC is of great significance for clinical applications.

BAG3, also named BIS or CAIR-1, is a multifunctional protein belonging to the family of BAG co-chaperones [10]. BAG3 protein primarily contains the BAG domain, WW domain, and a proline-rich (PXXP) domain, enabling it to participate in various chaperone or protein interactions [11]. The highly conserved BAG domain is located in the C-terminal region of the BAG3 protein, allowing it to bind to the ATPase domain of the HSC/HP70 chaperone and CRP78 and heat shock factor 1 [12–16]. The N-terminal WW domain of the BAG3 protein allows it to interact with various signaling and other polypeptides to regulate cellular adhesion, migration, and autophagy [17–21]. The PXXP domain of BAG3 can bind to proteins that have Src homology 3 (SH3), such as phospholipase C (PLC-) or Src, and it also serves as a docking station for the motor protein dynein [22–28].

In this research, we used TCGA and GEO databases to analyze the differential expression of BAG3 in KIRC versus normal kidney samples and to determine the relationship between its expression and clinicopathological features. Additionally, we identified differentially expressed genes of BAG3 in KIRC and investigated the possible biological functions and signaling pathways of these genes. Finally, we investigated the relationship between immune cell infiltration and differential BAG3 expression and the effect of BAG3 methylation on the prognosis of KIRC patients.

## Materials and methods 

### Data collection and processing

By utilizing TCGA database (https://portal.gdc.cancer.gov/), we obtained the RNA-Seq data and clinical data of 539 KIRC patients (Additional file 1: Table S1). The level 3 HTSeq-FPKM format data were converted into transcripts per million reads (TPM). The expression data were divided into high and low groups according to the median BAG3 expression level. In addition, the RNA-Seq data (GSE53757) were collected from GEO (http://www.ncbi.nlm.nih.gov/geo/)database, which included 72 tumor samples and their adjacent tissues, was used to verify the differential expression of BAG3.

### Pathological sample collection

The HKH cohort included 78 KIRC patients diagnosed at the Haikou Hospital Affiliated with Xiangya School of Medicine, Central South University, from 2015 to 2021. Patients underwent curative surgical treatment and received no chemotherapy or radiation therapy before surgery. The samples included 132 paraffin specimens (54 pairs of KIRC and the matched adjacent normal specimens and 24 KIRC tissue specimens). This research has been endorsed by ethics committee of Central South University Xiangya School of Medicine Affiliated Haikou Hospital and is based on the ethical requirements of the Helsinki Declaration. All participants have the right to know.

### Immunohistochemistry

IHC was used to examine BAG3 protein expression levels and distribution in paraffin-embedded tissue sections. After routine paraffin dewaxing to water, antigen repair was performed. After allowing it to cool naturally for 10 min, sections were washed thrice with PBS solution for 5 min each. Hydrogen peroxide solution (3%) was added to block endogenous peroxidase. Bovine serum albumin sealing solution (3%) was added, and the sections were incubated for 15 min. Next, the primary antibody against BAG3 (1:500 dilution; Proteintech, 10599-1-AP) was added, and the sections were incubated overnight at 4 °C. After that, they were washed thrice with the PBS solution, each time for 5 min. The secondary antibody (1:100 dilution) was added, and the sections were incubated for 50 min at room temperature. DAB was added for color development, after which the sections were re-dyed with hematoxylin for 3 min. Finally, the sections were subjected to dehydration until the sections became transparent, mounted with neutral gum, observed under a microscope, and photographed. The final IHC score was obtained by multiplying the staining intensity (SP) with the positive staining percentage (SI) of the cells. SI was scored as follows: 0: < 5%; 1: 5–25%; 2: 25–50%; 3: 51–75% and 4: 75–100%. SP was subjectively scored as follows: 0, no staining; 1, weak staining; 2, moderate staining; and 3, intense staining. Patients with a final IHC score ≥ 6 were included in the high BAG3 group, whereas those with a final IHC score < 6 were included in the low BAG3 group. Two independent pathologists who were blinded to the source of the slides examined and scored each sample [29].

### Identification of differentially expressed genes

R package DEseq2(1.26.0) [30]and Student’s t test were used to identify differentially expressed genes (DEGs) between high BAG3 expression and low BAG3 expression samples from the TCGA database. Adjusted *p* < 0.05 and |log2-fold change (FC)|> 1.0 were set as thresholds for the DEGs.

### Gene set enrichment analysis

The "clusterProfiler" R package [31, 32] was used to perform gene set enrichment analysis. The c2.cp.v7.0.symbols.gmt [Curated] in Molecular Signatures Database (MSigDB) collections was selected as a reference gene set. Gene sets with a false discovery rate (FDR) < 0.25 and adjusted *p* < 0.05 were considered significantly enriched.

### Gene–gene and protein–protein interaction networks analysis of BAG3

The gene and protein interaction networks of BAG3 were constructed using GeneMANIA database (http://www.genemania.org) [33] and STRING database (http://string-db.org) [34], respectively.

### DNA methylation analysis

Gene Set Cancer Analysis (GSCA; http://bioinfo.life.hust.edu.cn/GSCA/#/) database was utilized to analyze the relationship between BAG3 DNA methylation levels and its expression and prognostic significance in clear cell carcinoma [35].

### Correlation between BAG3 expression and immune infiltration

To determine the infiltration of immune cells in each sample, ssGSEA was performed using the GSVA package in R [36], and enrichment scores were obtained using particular gene markers for each kind of immune cell [37]. The Spearman’s correlation analysis was used to investigate the correlation between BAG3 expression and these immune cells.

### Construction and evaluation of the nomogram

To predict the overall survival probability, a nomogram was established based on independent prognostic factors in multivariate Cox analysis. Calibration plots were then used to assess the performance of the nomogram, and the concordance index (C-index) was used to quantify the discrimination of the nomogram [38]. The nomogram and calibration plots were created using the R package RMS (version 5.1–4) [39].

### Statistical analysis

BAG3 expression in unpaired and paired samples was analyzed using the Wilcoxon rank-sum test and matched samples *t*-test, respectively. In addition, the Kruskal–Wallis test, univariate regression Cox analysis, and multivariate Cox regression analysis were applied to investigate whether BAG3 expression was associated with clinicopathological factors. Using the K-M method and log-rank test, we compared the differences in 10-year OS, DSS, and PFI between patients with high BAG3 expression and those with low BAG3 expression in TCGA. In all studies, *p* < 0.05 (bilateral) was defined as statistically significant. All statistical analyses and plots were conducted using R (Version 3.6.3).

## Results

### Expression of BAG3 is downregulated in KIRC

**Fig. 1:**
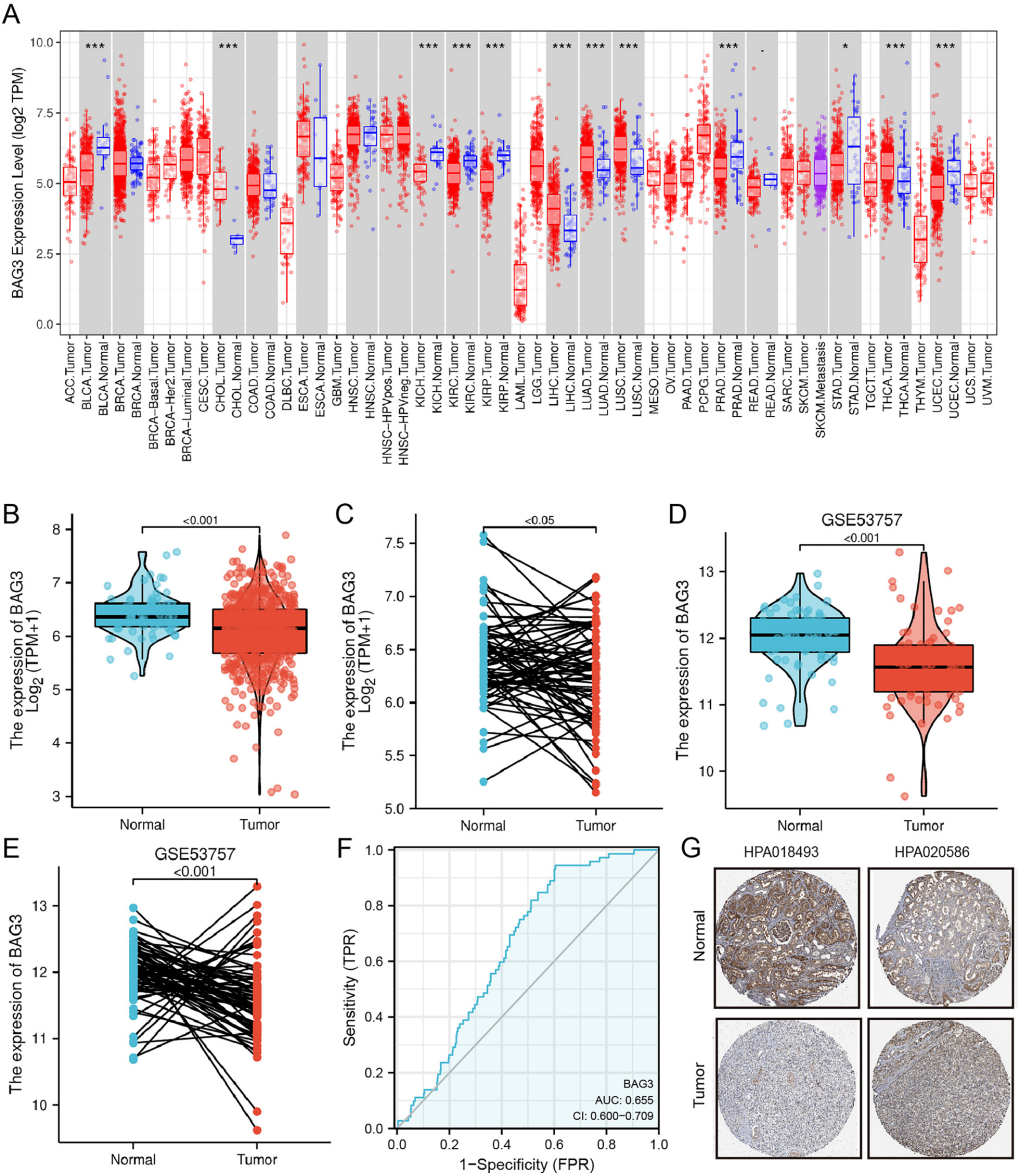
The expression level of BAG3 is downregulated in KIRC. **A** BAG3 expression levels in various cancers from TIMER2.0. **B**–**E** BAG3 expression in KIRC samples. **F** ROC curve was created to investigate the value of BAG3 in identifying KIRC tissues. **G** BAG3 protein level in KIRC from the HPA database

**Fig. 2:**
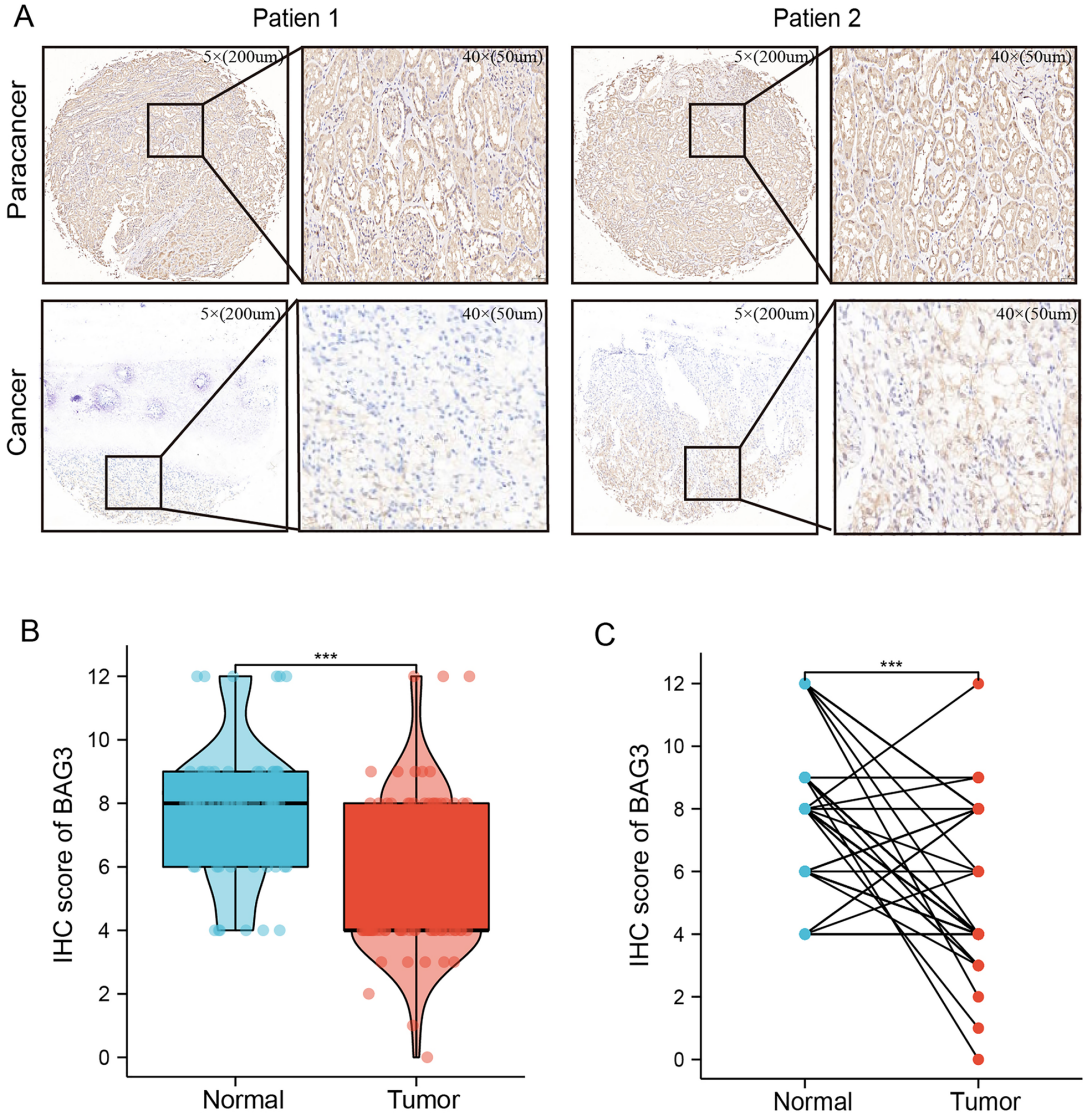
The verification of BAG3 expression in KIRC tissues and normal adjacent tissues using HKH cohort. **A** Representative images of IHC staining of BAG3 in KIRC tissues and matched normal tissues. The immunohistochemical assay was performed for detecting BAG3 expression. **B**, **C** The protein expression levels of BAG3 were significantly decreased in KIRC tissues compared with normal tissues; T-test was used for the statistical analysis. ***p < 0.001

The expression levels of BAG3 in various human cancers were evaluated according to the TIMER database. Compared with normal tissues, BAG3 was downregulated in kidney renal cell carcinoma, kidney renal papillary cell carcinoma, kidney chromophobe, bladder urothelial carcinoma, prostate adenocarcinoma, stomach adenocarcinoma, and uterine corpus endometrial carcinoma while BAG3 was upregulated in cholangiocarcinoma, liver hepatocellular carcinoma, lung adenocarcinoma, lung squamous cell carcinoma and thyroid carcinoma (Fig. 1A). We analyzed KIRC data sets from TCGA and GEO to investigate the differential expression of BAG3 in KIRC samples and normal samples. BAG3 expression in KIRC samples and normal tissues was analyzed using TCGA data. We found that compared with normal tissues, the expression level of BAG3 mRNA was considerably decreased in tumor tissues (Fig. 1B, C). Next, by using the GEO data, we further verified this result (Fig. 1D, E). Furthermore, we constructed the ROC curve indicating that BAG3 could be exploited as a potential biomarker with an area under the curve (AUC) of 0.655 (Fig. 1F). In addition, immunohistochemical analysis obtained by HPA showed that the expression of the BAG3 protein is downregulated in KIRC tissue compared to normal tissue (Fig. 1G). To further verify the protein expression level of BAG3 in KIRC, we used IHC to detect BAG3 protein expression in 78 cancer samples and 54 para-cancer samples (Additional file 2: Table S2). We selected two patients' cancer and para-cancer tissues as the reference samples (Fig. 2A). IHC results showed that the protein expression level of BAG3 was significantly downregulated in KIRC compared to normal tissues (*P* < 0.001) (Fig. 2B, C; Additional file 4 : Fig S1). These results demonstrated that BAG3 expression is downregulated at both the mRNA and protein levels in KIRC.

### Correlation between BAG3 expression and clinical characteristics

**Fig. 3:**
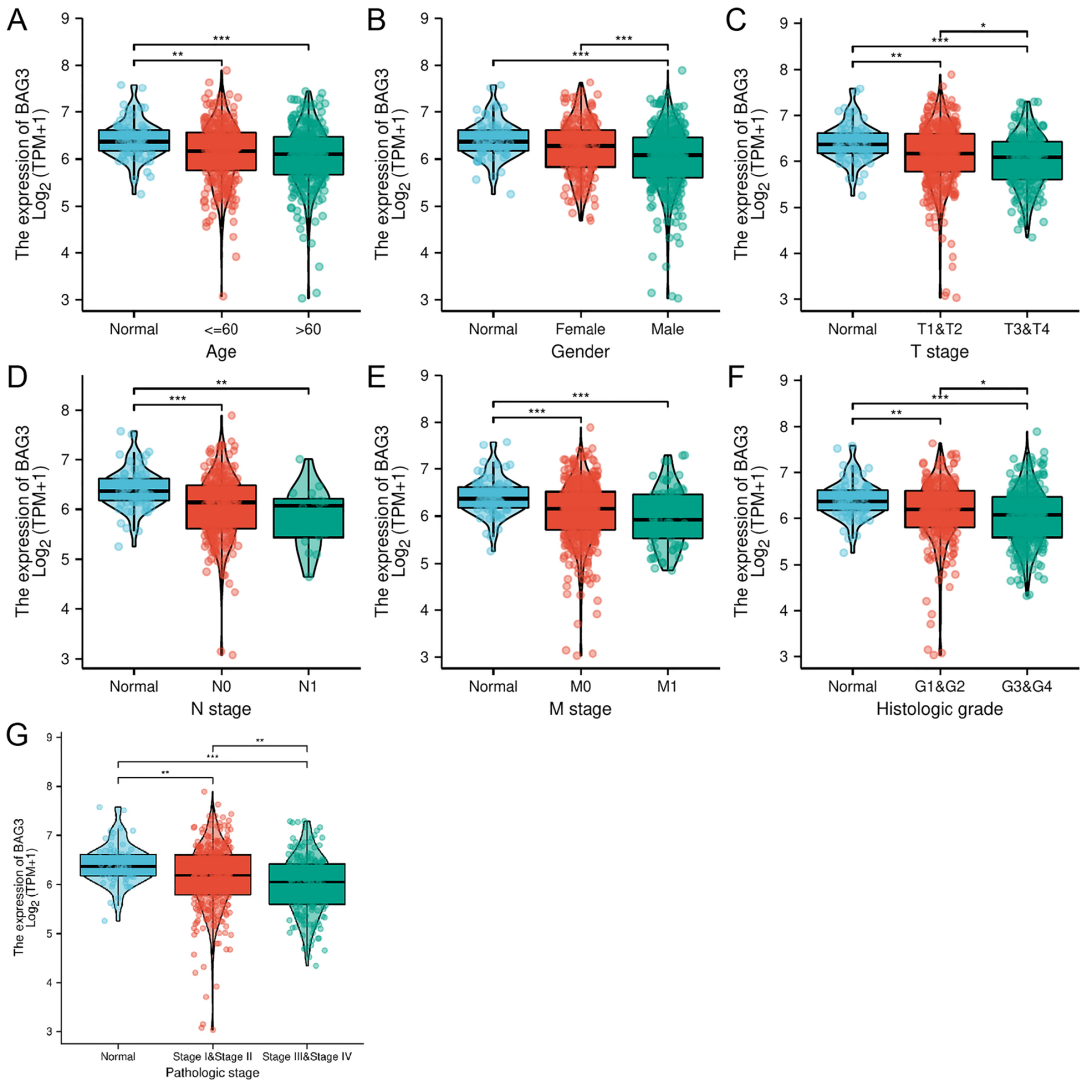
Correlation of BAG3 expression with clinicopathological characteristics. **A** Age. **B** Gender. **C** T stage. **D** N stage. **E** M stage. **F** Histologic grade. **G** Pathological stage. *p < 0.05, **p < 0.01, ***p < 0.001

To investigate the clinical characteristics of patients with different BAG3 expression levels, clinical data from KIRC patients in the TCGA database were acquired. The Kruskal–Wallis test was used to assess differences in clinicopathological variables after stratifying patients based on BAG3 expression, and the expression level of BAG3 was closely correlated with age, gender, TNM stage, histologic grade, and pathological stage (Fig. 3). Notably, a higher TNM grade, histological grade, and pathological stage were significantly associated with low BAG3 expression. Based on these results, patients with KIRC presenting lower BAG3 expression seemed to have a more advanced tumor stage.

### Correlation between BAG3 expression and prognosis

**Fig. 4:**
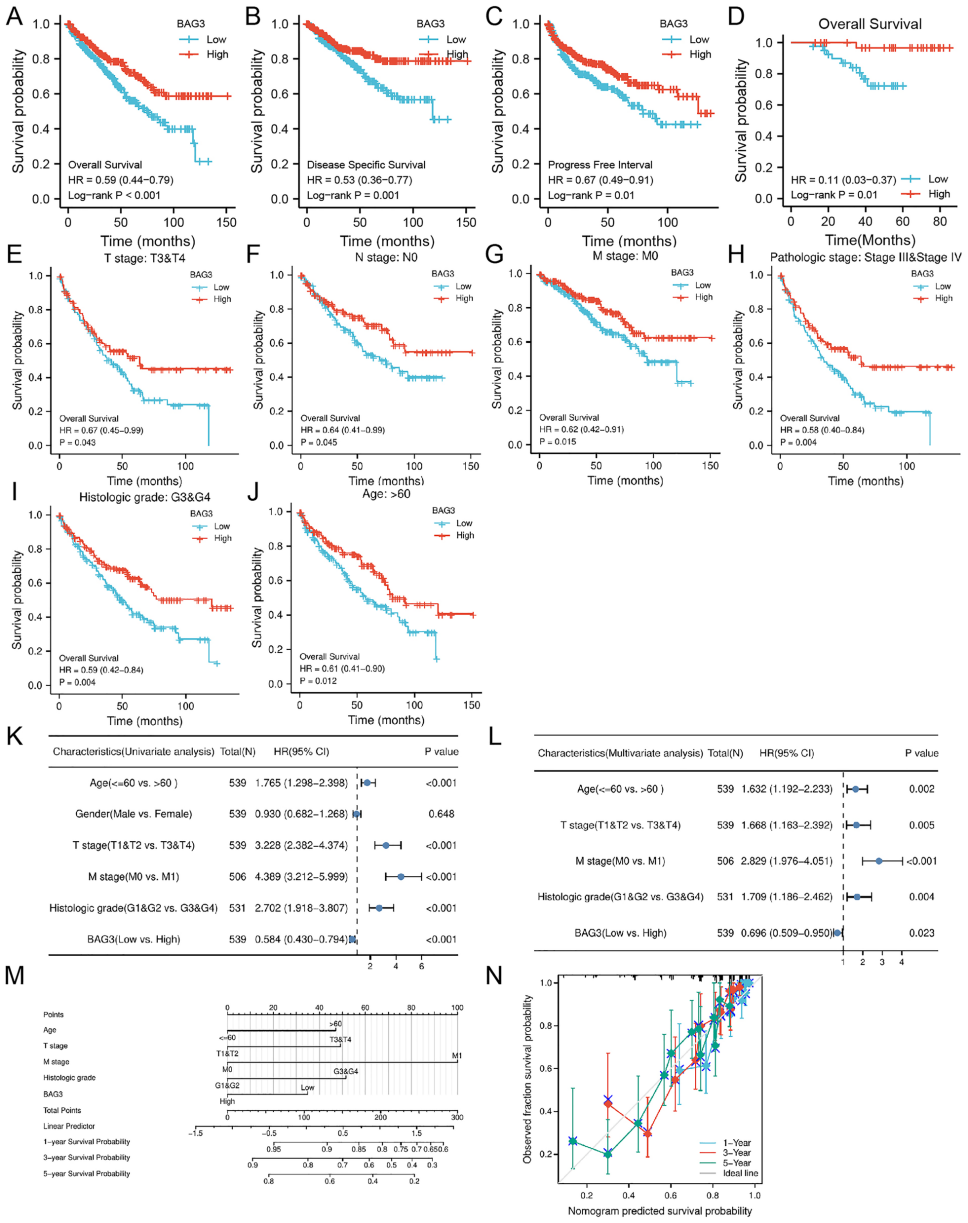
The prognostic value of BAG3 in KIRC. **A**–**C** Survival curves showing a comparison of OS, DSS and PFI between patients with KIRC presenting high and low BAG3 expression. **D** Survival curve showed that high BAG3 expression patients were associated with higher OS in HKH cohort. **E**–**J** OS survival curves of T3&4, N0, M0, stage III & IV, G3&4, and age > 60 subgroups between BAG3-high and -low patients with KIRC. **K**, **L** Univariate and multivariate Cox regression analyses. **M** For patients with KIRC, a nomogram was constructed to estimate the probability of 1-, 3-, and 5-year OS. **N** Nomogram calibration plots for determining the probability of OS at 1, 3, and 5 years

To investigate the correlation between BAG3 mRNA expression and the prognosis of KIRC patients, Kaplan–Meier curves with R package survminer and survival were used. Comparing the high BAG3 expression group, the OS, DSS, and PFI of the low BAG3 expression group exhibited a significantly worse prognosis (OS: hazard ratio [HR] = 0.58, 95% CI 1.65–3.25, *p* = 0.001; DSS: HR = 0.53, 95% CI 0.36–0.78, *p* = 0.001; PFI: HR = 0.67, 95% CI 0.49–0.91, *p* = 0.011) (Fig. 4A, B, C). The correlation between BAG3 expression and OS was further validated in the HKH cohort (Fig. 4D). Then, we investigated the associations between BAG3 expression and prognosis in several subgroups. In T3 and T4, N0, M0, Stage Ill and Stage IV, G3 and G4 and age over 60 subgroups. Patients in the BAG3 high expression group experienced better OS (Fig. 4E–J). To further identify factors associated with different prognoses, univariate and multivariate Cox regression analyses were performed with age, gender, TNM stage, histologic grade, and BAG3 expression levels. Univariate Cox regression analysis showed that low expression of BAG3, high T stage classification, distant metastasis, advanced age, and advanced histologic grade were significantly associated with poor OS (Fig. 4K; Table 1). Multivariate regression analysis confirmed that BAG3 expression, age, T stage, M stage, and histologic grade were independent prognostic factors for OS in KIRC patients (Fig. 4L). Additionally, we analyzed the risk factors of OS in 78 KIRC patients with univariate and multivariate Cox regression analysis. High T stage classification (*P* = 0.007) and low BAG3 expression (*P* = 0.023) were risk variables linked to poor outcomes for individuals with KIRC, according to multivariate Cox regression analysis (Table 2). According to the results of the multivariate Cox regression analysis, BAG3 expression and other independent clinicopathological factors were used to construct the point scale of the nomogram. Each variable was scored concerning the scale of the nomogram, and the survival probabilities of patients at 1, 3, and 5 years were predicted according to the total score. The C-index of the nomogram was 0.756 (95% confidence interval: 0.737–0.775). This result suggested that the prognostic nomogram of BAG3 had good discriminatory power (Fig. 4M). The calibration plot's bias-corrected line, almost parallel to the ideal curve (the 45-degree line), demonstrated good agreement between the forecast and the observation (Fig. 4N).

**Table 1:** Cox regression analysis of the association between BAG3 expression and clinical characteristics of TCGA cohort

Characteristics	Total (*N*)	Univariate analysis		Multivariate analysis	
		Hazard ratio (95% CI)	*P* value	Hazard ratio (95% CI)	*P* value
Age	539				
≤ 60	269	Reference			
> 60	270	1.765 (1.298–2.398)	** < 0.001**	1.632 (1.192–2.233)	**0.002**
Gender	539				
Female	186	Reference			
Male	353	0.930 (0.682–1.268)	0.648		
T stage	539				
T1&T2	349	Reference			
T3&T4	190	3.228 (2.382–4.374)	** < 0.001**	1.668 (1.163–2.392)	**0.005**
M stage	506				
M0	428	Reference			
M1	78	4.389 (3.212–5.999)	** < 0.001**	2.829 (1.976–4.051)	** < 0.001**
Histologic grade	531				
G1&G2	249	Reference			
G3&G4	282	2.702 (1.918–3.807)	** < 0.001**	1.709 (1.186–2.462)	**0.004**
BAG3	539				
Low	269	Reference			
High	270	0.584 (0.430–0.794)	** < 0.001**	0.696 (0.509–0.950)	**0.023**

**Table 2:** Cox regression analysis of the association between BAG3 expression and clinical characteristics of HKH cohort

Characteristics	Total (*N*)	Univariate analysis		Multivariate analysis	
		Hazard ratio (95% CI)	*P* value	Hazard ratio (95% CI)	*P* value
Age	78				
≤ 60	42	Reference			
> 60	36	0.344 (0.089–1.332)	0.122		
Gender	78				
Female	19	Reference			
Male	59	0.726 (0.154–3.420)	0.685		
T stage	78				
T1&T2	61	Reference			
T3&T4	17	4.842 (1.387–16.909)	0.013	6.052 (1.646–22.253)	0.007
Histologic grade	78				
G1&G2	38	Reference			
G3&G4	40	1.242 (0.359–4.299)	0.732		
BAG3	78				
Low	40	Reference			
High	38	0.106 (0.013–0.841)	0.034	0.089 (0.011–0.717)	0.023

### DNA methylation analysis of BAG3 in KIRC

**Fig. 5:**
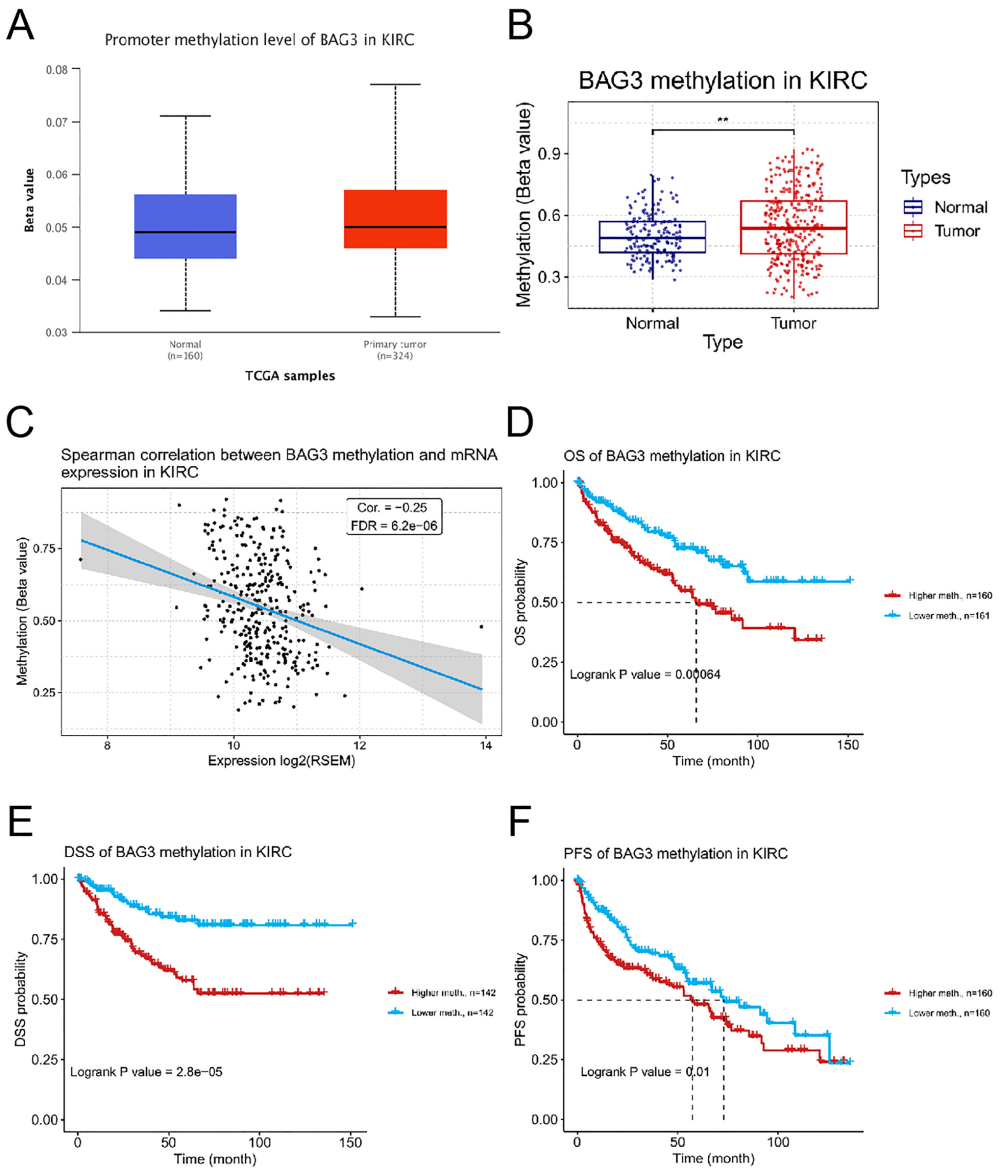
Correlation analysis between BAG3 methylation and KIRC patients based on ULCAN and GSCA database. **A** Mean methylation levels of the BAG3 promoter in KIEC versus normal tissues in TCGA cohort. **B** The methylation level of BAG3 between renal clear carcinoma tissues and normal renal tissues. **C** The correlation between NEIL3 methylation and mRNA expression. **D**–**F** The effect of NEIL3 methylation on overall survival (OS), progression-free survival (PFS), disease-specific survival (DSS), and disease-free interval (DFI) of patients with KIRC. **p < 0.01, ***p < 0.001

Previous research has found that abnormal DNA methylation is linked to the formation and progression of a variety of cancers [40, 41]. Using UALCAN and GSCA databases, we found that the methylation level of BAG3 in KIRC tissues was significantly higher than in normal tissues (Fig. 5A, B). Correlation analysis indicated that expression of BAG3 mRNA was considerably negatively correlated with its methylation status (cor = −0.25, FDR = 6.17e-6) (Fig. 5C). Furthermore, the BAG3 DNA methylation-high group was associated with poorer OS, DSS, and PFS as compared to the low group (Fig. 5D–F). These results suggest that DNA methylation of BAG3 might be involved in the development and progression of KIRC, which was closely related to the prognosis of patients with KIRC.

### Identification of DEGs in KIRC

**Fig. 6:**
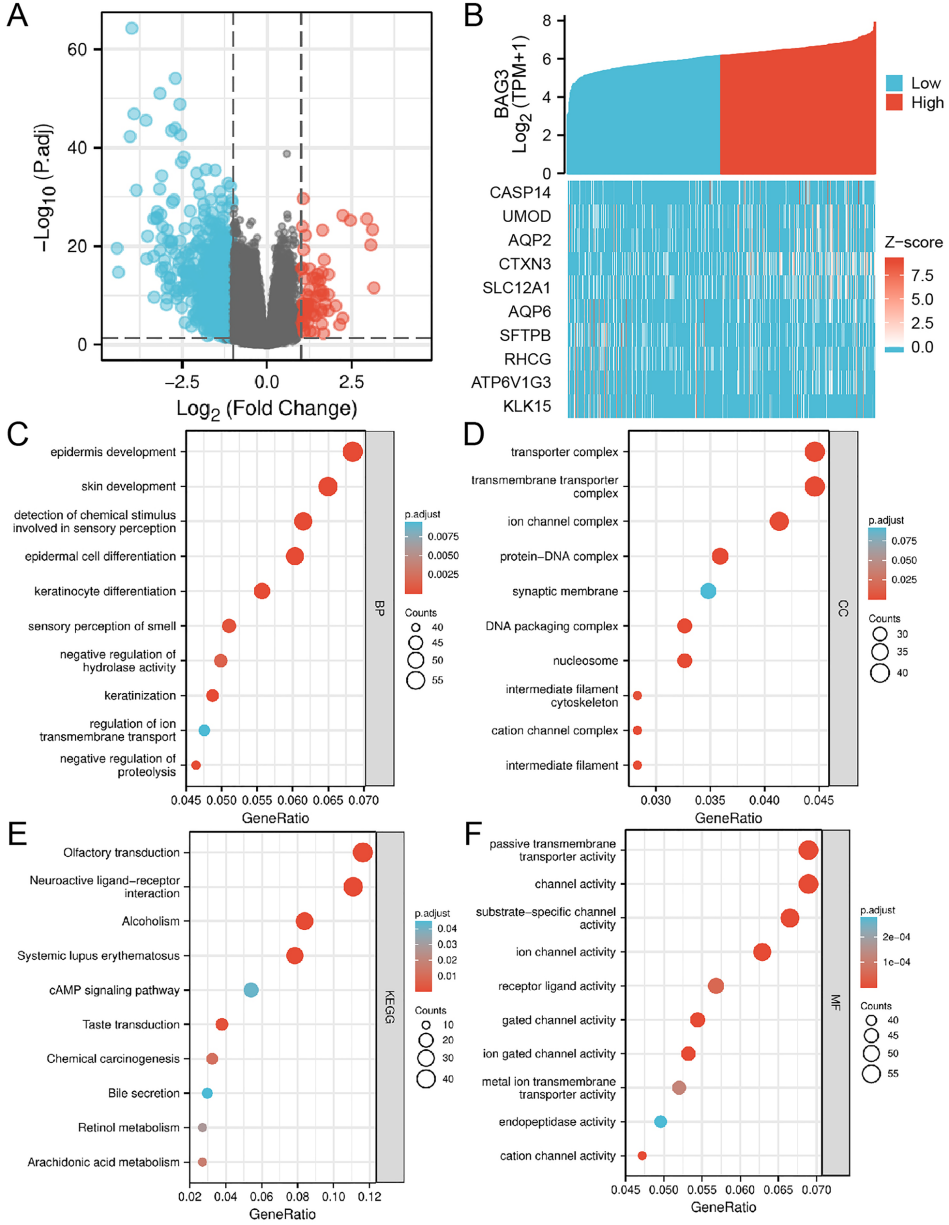
Functional enrichment analyses of BAG3-associated DEGs in KIRC. **A**, **B** Volcano plots of the DEGs and heatmap showing the top 10 DEGs. **C**–**F** GO and KEGG enrichment analyses based on BAG3-associated DEGs

Based on the median expression level of BAG3, we divided KIRC samples into high– and low–BAG3 expression groups. In total, 1030 DEGs were identified based on an analysis between the two groups using sequence data from TCGA. 65 DEGs were associated with the high high-BAG3 expression group, and 965 DEGs were associated with the low-BAG3 expression group (Fig. 6A). The top 10 DEGs were illustrated using a heatmap and sorted by relative expression (Fig. 6B).

### Functional enrichment analysis

**Fig. 7:**
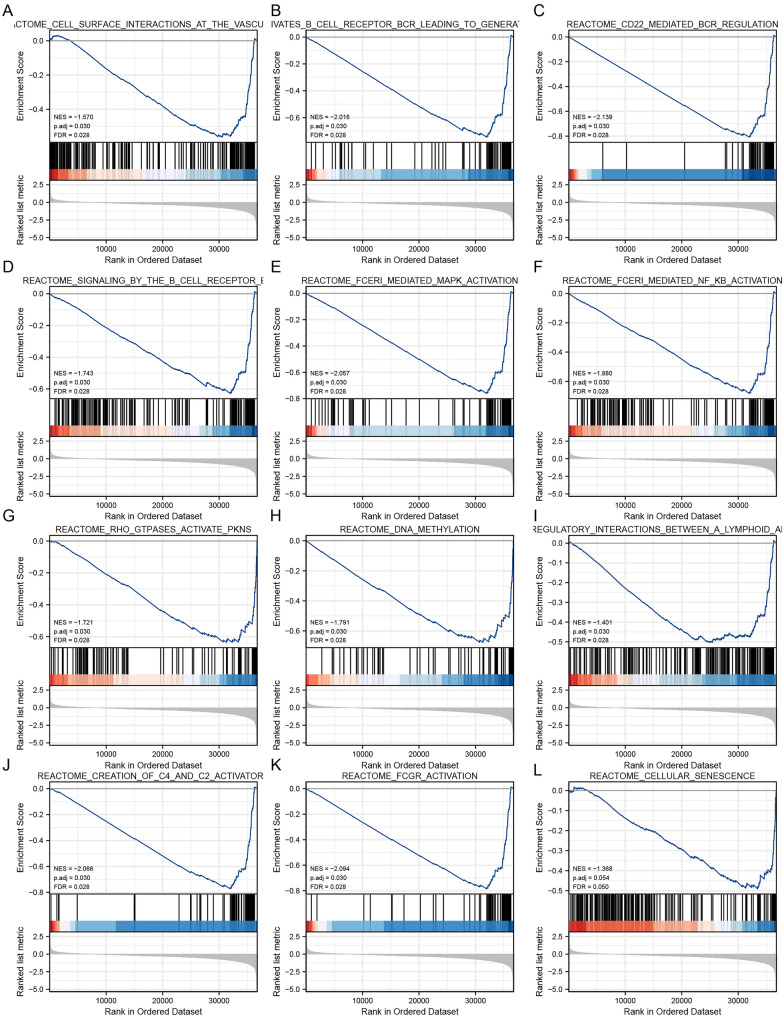
The gene set enrichment analysis of BAG3. **A**–**L** GSEA enrichment plots, including “Methylation”, “cellular senescence”, “Fc epsilon receptor (FceRI) signaling”, “FceRI-meditated MAPK activation”, “FceRI-mediated NF-κB activation”, “FCGR activation”, “immunoregulatory interactions between a lymphoid and a non-lymphoid cell”, “signaling by the B cell receptor”, “antigen activates B cell receptor leading to generation of second messengers”, “creation of C4 and C2 activators”, “CD22-mediated BCR regulation”, and “cell surface interactions at the vascular wall". NES, normalized enrichment score; p.adj, adjusted P-value; FDR, false discovery rate

Using the “clusterProfiler” R package to analyze the potential biological functions of BAG3-related DEGs, enriched consequences were ranked on the basis of the adjusted *p*-value. The following biological processes were significantly affected: epidermis development, skin development, epidermal cell differentiation, keratinocyte differentiation, etc. The terms of cell components are mainly enriched in transporter complex, transmembrane transporter complex, ion channel complex, protein–DNA complex, synaptic membrane, etc. Molecular functional primarily focus on passive transmembrane transporter activity, channel activity, etc. Additionally, KEGG pathway analysis showed that significantly DEGs-enriched pathways included olfactory transduction, neuroactive ligand–receptor interaction, alcoholism, systemic lupus erythematosus, cAMP signaling pathway, etc. (Fig. 6C–F). To further identify BAG3-related signaling pathways in KIRC, GSEA was conducted between the high and low BAG3 expression groups. “Methylation”, “cellular senescence”, “Fc epsilon receptor (FceRI) signaling”, “FceRI-meditated MAPK activation”, “FceRI-mediated NF-κB activation”, “FCGR activation”, “immunoregulatory interactions between a lymphoid and a non-lymphoid cell”, “signaling by the B cell receptor”, “antigen activates B cell receptor leading to generation of second messengers”, “creation of C4 and C2 activators”, “CD22-mediated BCR regulation”, and “cell surface interactions at the vascular wall” were significantly enriched in the group with low BAG3 expression (Fig. 7A–L). These results indicated that the gene sets specific to the high BAG3 expression group were mainly enriched in immune-associated pathways.

### Identification of BAG3-interacting genes and proteins

**Fig. 8:**
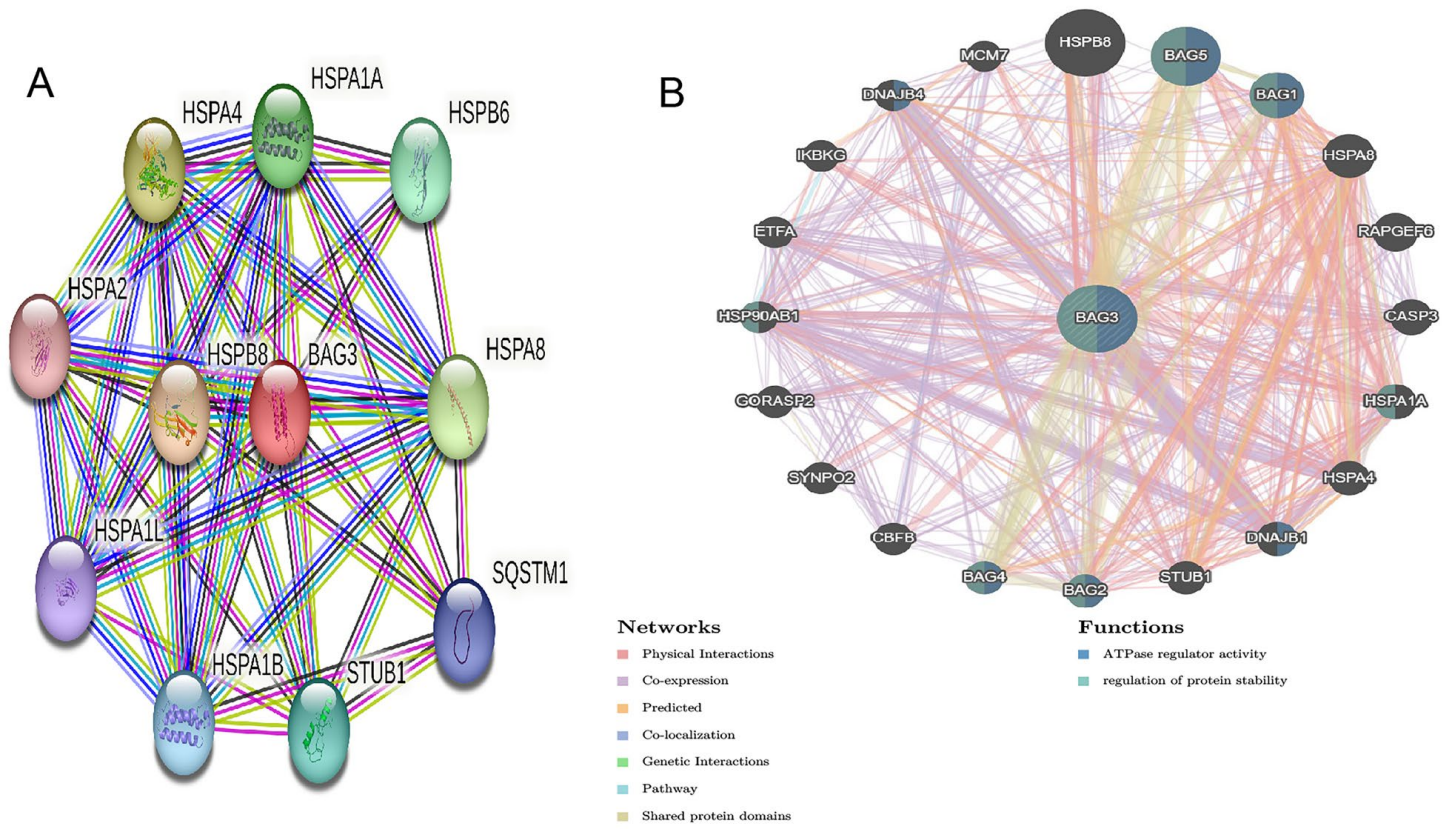
Analysis of gene–gene interaction (GGI) and protein–protein interaction (PPI) of BAG3. **A** The gene–gene interaction network of BAG3 was constructed using GeneMania. **B** The PPI network of BAG3 was generated using STRING

PPI network analysis of BAG3 was performed using the STRING database. The result showed that BAG3 was associated with HSPA4, HSPA8, HSPB8, HAPA1A, HSPB6, STUB1, HSPA1B, SQSTM1, HSPA2 and HSOA1L (Fig. 8A). The combined scores were 0.999, 0.999, 0.999, 0.998, 0.993, 0.988, 0.985, 0.98, 0.974, and 0.974, respectively. The gene–gene crosstalk network for BAG3 and the modified adjacent genes was constructed via GeneMania. It showed that the 20 genes were closely correlated with BAG3. Functional analysis suggested that these genes were strongly associated with ATPase regulator activity and regulation of protein stability (Fig. 8B).

### BAG3 expression was correlated with immune infiltration

**Fig. 9:**
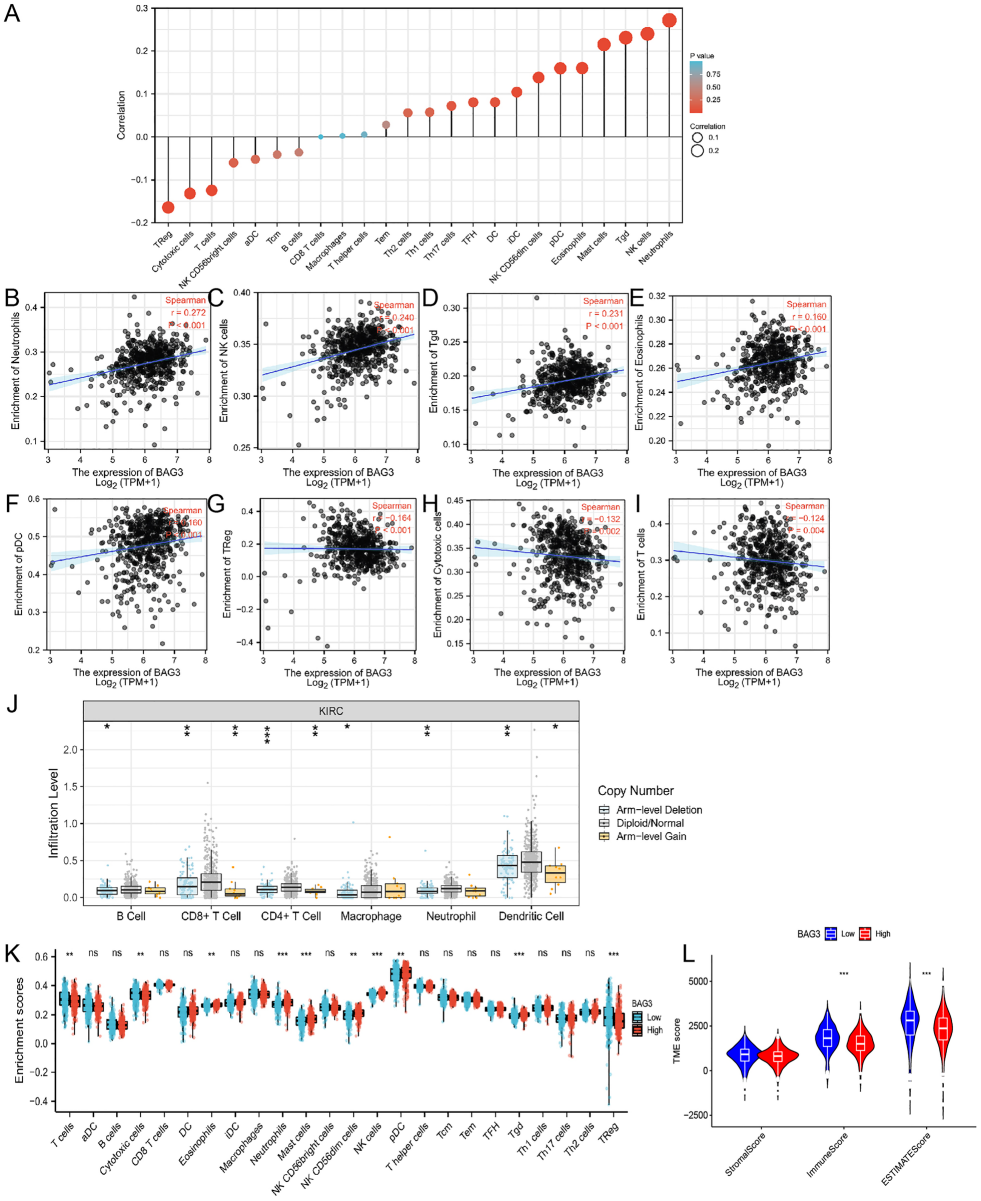
Integrative analysis of BAG3 expression in the infiltrating immune microenvironment. **A** The forest plot depicts the relationship between the level of BAG3 expression and the relative abundances of 24 immune cells. **B**–**I** Scatter plots showing the differentiation of neutrophils, NK cells, Tgd, eosinophils, neutrophils, pDC, TReg, cytotoxic cells and T cells infiltration levels between high and low groups of BAG3 expression.** J** The correlation of BAG3 somatic copy number alterations with immune cell infiltration.** K** Scatter plots showing the correlations between 24 immune cells and BAG3 expression levels.** L** The immuneScore, stromalScore, and ESTIMATEScore between high and low BAG3 group. *p < 0.05, **p < 0.01, ***p < 0.001, NS, no significance

With the ssGSEA algorithm, we analyzed the correlations between BAG3 expression and immune cell infiltration levels (Fig. 9A). The expression of BAG3 was negatively correlated with Treg, cytotoxic cells, T cells, CD56bright cells, aDCs, and Tcm cells and positively connected with Neutrophils, NK cells, Tgd cells, Mast cells, eosinophils, pDCs, NK CD56dim cells, iDCs, DCs, TFH, Th17 cells, Th1 cells, TH2 cells, Tems, T helper cells, macrophages, and CD8 T cells (Fig. 9B–I). We also use the TIMER database to investigate the link between BAG3 expression and tumor-infiltrating immune cell gene markers in KIRC (Additional file 3: Table S3). Tumor purity is an important aspect affecting the dissection of immune infiltration in clinical cancer biopsies. BAG3 expression was substantially correlated with the majority of immune markers in various types of immune cells in KIRC after correcting for tumor purity. This analysis of immune markers of different functions T cells showed that BAG3 expression was highly correlated with most immunomarkers (CD8A, CD8B, CD3D, CD3E, CD2, IFN-γ, IL12A, IL12B, STAT6, BCL6, STAT3, FOXP3, STAT5B, TGFβ, LAG3, CTLA4, TIM3, and GZMB) of T cells in KIRC. These results suggest that BAG3 may be critical in the T cells’ immune response to KIRC. Furthermore, the results also showed a significant correlation between BAG3 expression and the immunomarkers INOS and IRF5 of M1 macrophages in KIRC. It indicated that BAG3 may induce macrophages to M1 polarization in KIRC. The somatic copy number alteration (SCNA) module revealed that arm-level deletion of BAG3 was significantly associated with immune cell infiltration levels in KIRC (Fig. 9J). Subsequently, KIRC samples of TCGA were divided into BAG3-high and BAG3-low expression groups based on BAG3 expression levels to determine whether different BAG3 expression groups differ in the tumor immune microenvironment of KIRC (Fig. 9K). In the BAG3 high expression group, we found that Eosinophils, Neutrophils, NK CD56dim cells, NK cells, pDC, and Tgd cells increased (*P* < 0.05), while T cells, Cytotoxic cells, and Treg cells decreased (*P* < 0.05), Furthermore, in the TME scores obtained by the ESTIMATE algorithm. We found that ImmuneScore and ESTIMATEScore of the BAG3 low expression group was significantly higher than that of the BAG3 high expression group (Fig. 9L). These results further support that BAG3 expression is closely related to immune cell infiltration and suggest that BAG3 plays a significant role in the immune microenvironment of the KIRC.

**Fig. 10:**
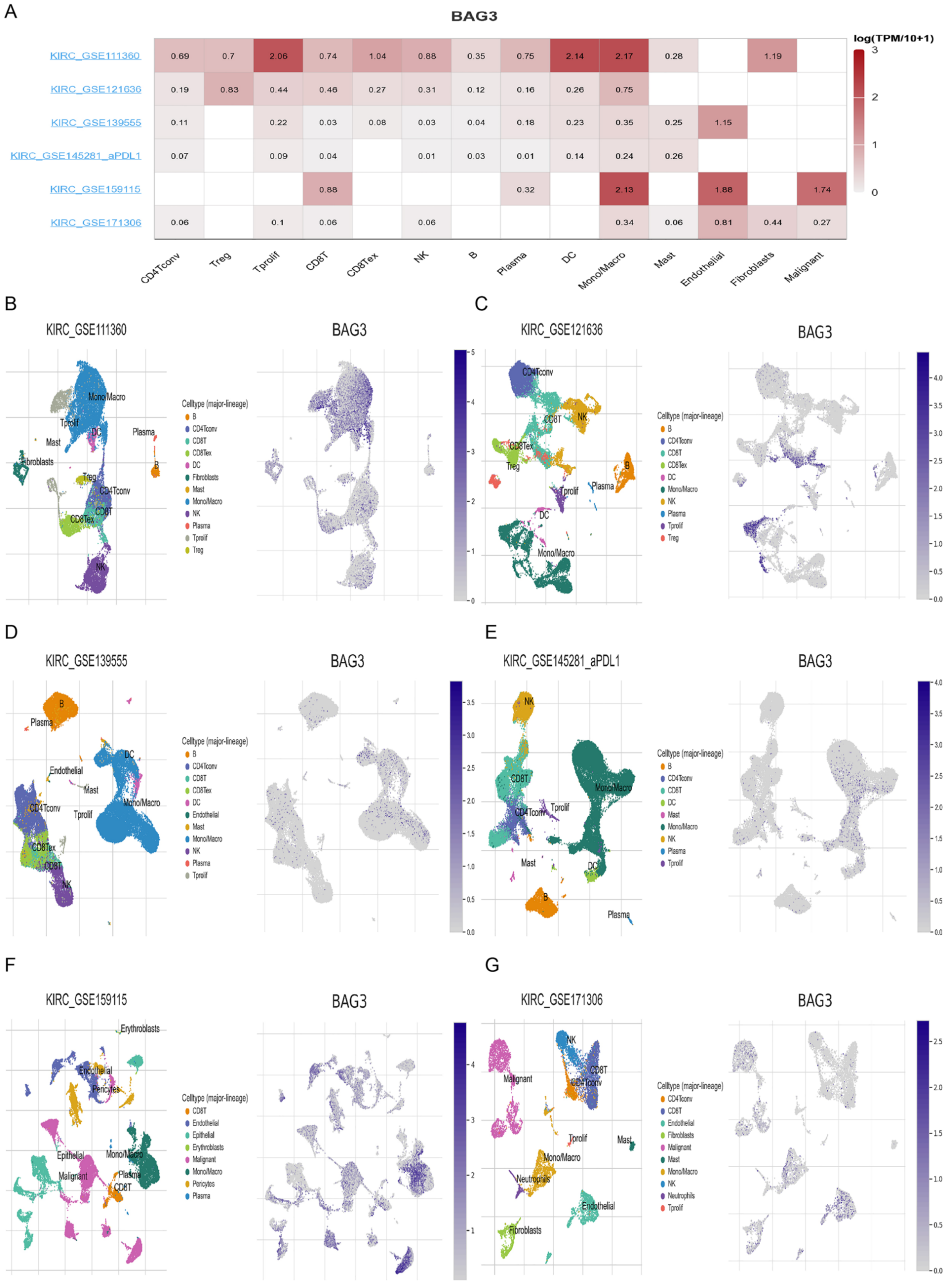
BAG3 at single-cell level. **A** Heatmap demonstrates BAG3 expression in cells from a variety of databases. **B**–**G** BAG single-cell cluster in different databases

To further validate the correlation between BAG3 and immune infiltration in gliomas, we analyzed single-cell sequencing datasets of the KIRC from the TISCH database. In the distribution heatmap (Fig. 10A), we found low to moderate BAG3 expression in immune cells (e.g., B cells, natural killer T cells, CD8 T cells, CD4 T cells, Plasma Cells, Tregs, and dendritic cells). BAG3 was primarily expressed at the macrophages cluster. We then analyzed the above datasets using single-cell cluster map, which were divided into various types of cells (Fig. 10B–G). BAG3 expression level remained the highest in macrophages, consistent with the results shown in Fig. 10A. Accordingly, the BAG3 expression level was quite different in distinct cell types, with the highest levels in macrophages instead of KIRC cells, suggesting that BAG3 may also play a role in immune cells besides cancer cells. The widespread expression of BAG3 in different kinds of immune cells confirms that it may have potential functions in the TME of KIRC (Additional file 4).

## Discussion

Kidney cell cancer is one of the deadliest urologic tumors. At the time of initial diagnosis, up to one in three patients already harbor distant metastases, leading to a poor prognosis [42]. With recent advancements in tumor detection, multiomics techniques in oncology research have seen significant growth [43]. The diagnosis and treatment of kidney cancer have greatly benefited from the combined use of artificial intelligence (AI) and radiogenomics [44, 45]. A study assessed the potential value in terms of mutation status of the gene encoding polybrominated-1 protein (PBRM1) in patients with renal clear cell carcinoma by high-dimensional quantitative CT texture analysis based on machine learning (ML) [46]. Radiomics analysis combined with machine learning provides the opportunity to non-invasively identify imaging features that can predict prognostic genomic and histopathologic indicators, which is expected to guide our subsequent clinical translation of KIRC biomarker BAG3 through radiogenomic. BAG3 has been implicated in various human diseases, including cancer, myopathies, and nervous system diseases [47–49]. Previous research has reported that BAG3 is associated with adverse prognoses in a variety of tumors, such as pancreatic cancer [50], Colon Cancer [51], endometrioid endometrial adenocarcinoma [52], and glioblastoma [53]. However, its role and mechanism in KIRC are still unclear.

In the present study, we discovered that the expression of BAG3 in KIRC was significantly lower than that in normal kidney tissue by analyzing the TCGA and GEO database. The IHC results further validated the downregulation of BAG3 in KIRC. The ROC curve analysis indicates that BAG3 expression may be a potential diagnostic biomarker in distinguishing KIRC from normal tissue. Moreover, we investigated the association between BAG3 expression and clinical data and prognosis. It was found that decreased BAG3 expression was associated with unfavorable prognosis and poor clinicopathological characteristics, including high T stage, high histologic grade, advanced pathological stage, and distant metastasis. Both Kaplan–Meier survival analyses and univariate and multivariate Cox regression analyses consistently demonstrated that low BAG3 expression in KIRC was strongly associated with poor patient prognosis. Then, we constructed a nomogram that combines BAG3 expression with clinical data. With a good C-index of 0.756 (0.737–0.775), this nomogram predicted the 1-, 3-, and 5-year OS of patients with KIRC with high accuracy.

In addition, we further identified DEGs associated with BAG3 to explore the potential functions of BAG3 in KIRC. By using GO and KEGG enrich analyses, we found that DEGs were mainly concentrated in epidermis development, cell differentiation, keratinization, metal ion transmembrane transporter activity, channel activity, Systemic lupus erythematosus, cAMP signaling pathway, etc. The results of GSEA showed that in the low-expression BAG3 phenotype, pathways such as "methylation", "FceRI-mediated NF-κB activation", "FceRI-mediated MAPK activation", "FCGR activation", "immunoregulatory interactions between a lymphoid and a non-lymphoid cell", "signaling by the B cell receptor", and "CD22-mediated BCR regulation" were significantly enriched. Previous studies have shown that FCGR-mediated ADCC (Antibody-dependent cellular cytotoxicity) plays a critical role in anti-tumor immunity [54–56]. Several studies have found that the NF-κB pathway is crucial in targeted therapy and immune response in KIRC [57–60]. In addition, Massimo et al [61]. found involvement of the NF-κB pathway in BAG3-mediated modulation of apoptosis. Huang et al. [62] found that inhibition of MAPK signaling pathways may inhibit renal cell carcinoma growth by disrupting tumor vasculature. Tie et al. [63] demonstrated that activation of the cAMP signaling pathway enhanced the ability of renal cancer cells to proliferate and migrate. BAG3 interacts with the SH3 domain-containing PLCG1 protein by the PXXP motif [27]. This interaction may affect the stability and activity of PLCG1, thereby regulating its role in the FGF signaling pathway. The FGF/FGFR signaling pathway is critical in tumor cells' growth, differentiation, and survival [64]. Therefore, understanding the specific mechanisms of BAG3 and PLCG1 interaction may provide valuable information for developing new anticancer treatment strategies. The immunoregulatory interactions between a lymphoid and a non-lymphoid cell pathway play a crucial role in modifying the response of cells of lymphoid origin (such as B, T and NK cells) to self and tumor antigens, as well as to pathogenic organisms [65, 66]. Taken together, BAG3 may be involved in these immune-related signaling pathways to regulate the immune microenvironment and immune response, influencing the development and progression of KIRC.

Tumor-infiltrating lymphocytes (TILs), an important component of the tumor microenvironment (TME), were recently shown to play a pivotal regulatory role in the occurrence and development of tumors [67]. Next, we explored the relationship between BAG3 and immune cell infiltration in KIRC. We found that BAG3 expression was clearly associated with the infiltration of Treg cells, T cells, DCs, neutrophils, NK cells, and mast cells. A previous study showed that the subsets of KIRC with the highest T cell accumulation have the worst survival rates [68]. Treg cells infiltrate heavily in the tumor microenvironment, not only inhibiting tumor immunity but also promoting tumor proliferation, invasion, and metastasis [69]. Multiple studies reported increased Treg cell infiltration associated with poor prognosis in ccRCC [70, 71]. A previous study suggested that mast cells infiltration may have a protective effect on renal cell carcinoma [72]. Dendritic cells (DCs) are the most potent antigen-presenting cells (APCs) able to activate naive T cells and then initiate anti-tumor immune responses [73]. NK cells are essential in the anti-tumor immune response through interaction with DCs [74]. NK cells have been shown to exhibit anti-tumor cytotoxicity against various malignancies [75]. In addition, we found that BAG3 expression is strongly correlated with various immunomarker groups in KIRC. Thus, we speculated that BAG3 may affect the prognosis of KIRC patients by regulating the degree of immune cell infiltration.

DNA methylation is the main mode of epigenetic modification [76]. Hypermethylation of the promoter region leads to transcriptional repression of the tumor suppressor gene [77]. Our study showed that the methylation level of the BAG3 promoter was significantly elevated in KIRC, while BAG3 expression was markedly lower in KIRC than in normal tissues. These results suggest that the low BAG3 expression in KIRC may be due to hypermethylated methylation of BAG3. In addition, we found that methylation of BAG3 was associated with the clinical prognosis of KIRC patients, and patients with hypomethylated BAG3 had worse OS, DSS, and PFS.

However, there were some limitations in this study. The research on BAG3 in KIRC is still in its infancy, and our study is restricted to the analysis of bioinformatics databases and experimental confirmation of IHC. Therefore, further in vivo and in vitro experiments are necessary to elucidate BAG3's detailed molecular mechanisms in KIRC. As a whole, we have integrated BAG3 expression levels with clinical characteristics to construct a comprehensive nomogram that can effectively guide clinical practice. The findings from our study could provide valuable insights for personalized treatment and patient management, thereby enhancing prognostic assessments for KIRC patients.

## Conclusion

In summary, using bioinformatics analysis techniques and multiple databases, this study is the first comprehensive analysis of BAG3 in KIRC. Our study suggested that the downregulated of BAG3 expression in KIRC was significantly associated with poor prognosis. BAG3 might affect the development and progression of KIRC by regulating multiple immune-related signaling pathways. In addition, BAG3 expression was correlated with multiple immune cells and affects the immune cell infiltration of KIRC. These results suggested that BAG3 may be a novel potential prognostic and immune-associated biomarker for KIRC patients.

### Supplementary Information

**Additional file 1: Table S1.** Characteristics of patients with KIRC in TCGA.

**Additional file 2: Table S2.** Characteristics of patients with KIRC in HKH cohort.

**Additional file 3: Table S3.** Correlation analysis between BAG3 expression and biomarkers of immune cells.

**Additional file 4. Fig S1.** Tissue microarray.

## Data Availability

The datasets analyzed during this study are available in the TCGA repository (https://portal.gdc.cancer.gov/) and the GEO database (https://www.ncbi.nlm. nih.gov/geo/query/acc.cgi?acc = gse53757).
