# Supplementary material for: BAG3 as a novel prognostic biomarker in kidney renal clear cell carcinoma correlating with immune infiltrates
Source: Eur J Med Res. 2024 Feb 1;29:93. doi: 10.1186/s40001-024-01687-w (PMC10832118; doi:10.1186/s40001-024-01687-w)
Supplement: Supplementary file 1 — Additional file 1: Table S1. Characteristics of patients with KIRC in TCGA. [file 40001_2024_1687_MOESM1_ESM.docx]

**Supplementary Table 1** Characteristics of patients with KIRC in TCGA.

| Characteristic | levels | Overall |
| --- | --- | --- |
| n |  | 539 |
| Age, n (%) | <=60 | 269 (49.9%) |
|  | >60 | 270 (50.1%) |
| Gender, n (%) | Female | 186 (34.5%) |
|  | Male | 353 (65.5%) |
| T stage, n (%) | T1 | 278 (51.6%) |
|  | T2 | 71 (13.2%) |
|  | T3 | 179 (33.2%) |
|  | T4 | 11 (2%) |
| N stage, n (%) | N0 | 241 (93.8%) |
|  | N1 | 16 (6.2%) |
| M stage, n (%) | M0 | 428 (84.6%) |
|  | M1 | 78 (15.4%) |
| Pathologic stage, n (%) | Stage I | 272 (50.7%) |
|  | Stage II | 59 (11%) |
|  | Stage III | 123 (22.9%) |
|  | Stage IV | 82 (15.3%) |
| Histologic grade, n (%) | G1 | 14 (2.6%) |
|  | G2 | 235 (44.3%) |
|  | G3 | 207 (39%) |
|  | G4 | 75 (14.1%) |
| Age, median (IQR) |  | 61 (52, 70) |
| Characteristic | levels | Overall |
| n |  | 539 |
| Age, n (%) | <=60 | 269 (49.9%) |
|  | >60 | 270 (50.1%) |
| Gender, n (%) | Female | 186 (34.5%) |
|  | Male | 353 (65.5%) |
| T stage, n (%) | T1 | 278 (51.6%) |
