# Supplementary material for: BAG3 as a novel prognostic biomarker in kidney renal clear cell carcinoma correlating with immune infiltrates
Source: Eur J Med Res. 2024 Feb 1;29:93. doi: 10.1186/s40001-024-01687-w (PMC10832118; doi:10.1186/s40001-024-01687-w)
Supplement: Supplementary file 2 — Additional file 2: Table S2. Characteristics of patients with KIRC in HKH cohort. [file 40001_2024_1687_MOESM2_ESM.docx]

**Supplementary Table 2** Characteristics of patients with KIRC in HKH cohort.

| Characteristic | levels | Overall |
| --- | --- | --- |
| n |  | 78 |
| Age, n (%) | <=60 | 42 (53.8%) |
|  | >60 | 36(46.2%) |
| Gender, n (%) | Female | 19 (24.4%) |
|  | Male | 59 (75.6%) |
| T stage, n (%) | T1 | 49 (62.8%) |
|  | T2 | 12 (15.5%) |
|  | T3 | 14 (17.9%) |
|  | T4 | 3 (3.8%) |
| N stage, n (%) | N0 | 77 (98.7%) |
|  | N1 | 1 (1.3%) |
| M stage, n (%) | M0 | 75 (96.2%) |
|  | M1 | 3 (3.8%) |
| Histologic grade, n (%) | G1 | 11 (14.1%) |
|  | G2 | 27 (34.6%) |
|  | G3 | 33 (42.3%) |
|  | G4 | 7 (9.0%) |
| Age, median (IQR) |  | 77 (40, 74) |
