# Supplementary material for: BAG3 as a novel prognostic biomarker in kidney renal clear cell carcinoma correlating with immune infiltrates
Source: Eur J Med Res. 2024 Feb 1;29:93. doi: 10.1186/s40001-024-01687-w (PMC10832118; doi:10.1186/s40001-024-01687-w)
Supplement: Supplementary file 3 — Additional file 3: Table S3. Correlation analysis between BAG3 expression and biomarkers of immune cells. [file 40001_2024_1687_MOESM3_ESM.docx]

**Supplementary Table 3** Correlation analysis between BAG3 expression and biomarkers of immune cells.

| **Description** | **Gene marker** | **None** | | **Purity** | |
| --- | --- | --- | --- | --- | --- |
|  |  | cor | p | cor | p |
| CD8+ T cell | **CD8A** | -0.082 | 0.057 | -0.121 | **0.009** |
|  | **CD8B** | -0.089 | **0.039** | -0.130 | **0.005** |
| T cell (general) | **CD3D** | -0.156 | **0.000** | -0.206 | **0.000** |
|  | **CD3E** | -0.097 | **0.025** | -0.136 | **0.003** |
|  | **CD2** | -0.130 | **0.003** | -0.171 | **0.000** |
| B cell | **CD19** | -0.109 | **0.012** | -0.117 | **0.012** |
|  | CD79A | -0.084 | 0.053 | -0.110 | **0.019** |
| Monocyte | CD86 | -0.021 | 0.622 | -0.069 | 0.141 |
|  | CD115(CSF1R) | 0.042 | 0.333 | 0.010 | 0.823 |
| TAM | **CCL2** | 0.235 | **0.000** | 0.250 | **0.000** |
|  | CD68 | -0.004 | 0.931 | -0.038 | 0.420 |
|  | IL10 | 0.019 | 0.656 | -0.034 | 0.469 |
| M1 Macrophage | **INOS(NOS2)** | 0.408 | **0.000** | 0.401 | **0.000** |
|  | **IRF5** | -0.172 | **0.000** | -0.171 | **0.000** |
|  | COX2(PTGS2) | 0.065 | 0.136 | 0.055 | 0.240 |
| M2 Macrophage | **CD163** | 0.134 | **0.002** | 0.085 | 0.067 |
|  | VSIG4 | -0.006 | 0.894 | -0.068 | 0.144 |
|  | MS4A4A | 0.036 | 0.401 | -0.006 | 0.906 |
| Neutrophils | CD66b(CEACAM8) | 0.014 | 0.747 | 0.000 | 0.998 |
|  | CD11b(ITGAM) | 0.031 | 0.474 | -0.007 | 0.884 |
|  | CCR7 | 0.033 | 0.449 | 0.011 | 0.811 |
| Natural killer cell | **KIR2DL1** | 0.147 | **0.001** | 0.129 | **0.006** |
|  | **KIR2DL3** | 0.108 | **0.013** | 0.067 | 0.149 |
|  | KIR2DL4 | 0.044 | 0.309 | -0.003 | 0.941 |
|  | **KIR3DL1** | 0.158 | **0.000** | 0.117 | **0.012** |
|  | KIR3DL2 | 0.077 | 0.075 | 0.072 | 0.122 |
|  | KIR3DL3 | 0.056 | 0.199 | 0.054 | 0.249 |
|  | **KIR2DS4** | 0.159 | **0.000** | 0.151 | **0.001** |
| Dendritic cell | HLA-DPB1 | 0.008 | 0.848 | -0.036 | 0.436 |
|  | HLA-DQB1 | 0.010 | 0.815 | -0.008 | 0.869 |
|  | HLA-DRA | 0.005 | 0.903 | -0.044 | 0.347 |
|  | HLA-DPA1 | 0.019 | 0.666 | -0.016 | 0.735 |
|  | BDCA-1(CD1C) | 0.069 | 0.111 | 0.051 | 0.271 |
|  | **BDCA-4(NRP1)** | 0.455 | **0.000** | 0.445 | **0.000** |
|  | **CD11c(ITGAX)** | -0.098 | **0.023** | -0.113 | **0.015** |
| Th1 | T-bet(TBX21) | 0.053 | 0.221 | 0.036 | 0.435 |
|  | STAT4 | -0.055 | 0.206 | -0.070 | 0.134 |
|  | STAT1 | 0.073 | 0.091 | 0.036 | 0.442 |
|  | **IFN-γ（IFNG）** | -0.181 | **0.000** | -0.229 | **0.000** |
|  | TNF-α(TNF) | -0.025 | 0.567 | -0.060 | 0.195 |
| Th2 | **IL12A** | 0.109 | **0.012** | 0.082 | 0.077 |
|  | **IL12B** | -0.137 | **0.001** | -0.112 | **0.016** |
|  | GATA3 | -0.024 | 0.574 | -0.040 | 0.386 |
|  | **STAT6** | 0.228 | **0.000** | 0.235 | **0.000** |
|  | STAT5A | 0.055 | 0.203 | 0.027 | 0.570 |
|  | IL13 | -0.040 | 0.357 | -0.029 | 0.539 |
| Tfh | **BCL6** | 0.276 | **0.000** | 0.270 | **0.000** |
|  | IL21 | -0.054 | 0.215 | -0.082 | 0.078 |
| Th17 | **STAT3** | 0.494 | **0.000** | 0.472 | **0.000** |
|  | IL17A | -0.079 | 0.068 | -0.061 | 0.190 |
| Treg | **FOXP3** | -0.171 | **0.000** | -0.198 | **0.000** |
|  | CCR8 | -0.065 | 0.136 | -0.078 | 0.093 |
|  | **STAT5B** | 0.410 | **0.000** | 0.401 | **0.000** |
|  | **TGFβ(TGFB1)** | 0.214 | **0.000** | 0.190 | **0.000** |
| T cell exhaustion | **LAG3** | -0.163 | **0.000** | -0.282 | **0.000** |
|  | **CTLA4** | -0.098 | **0.024** | -0.278 | **0.000** |
|  | **TIM3(HAVCR2)** | 0.098 | **0.024** | -0.138 | **0.003** |
|  | **GZMB** | 0.006 | 0.898 | -0.262 | **0.000** |
|  | **PD-1(PDCD1)** | -0.122 | **0.005** | -0.265 | **0.000** |
