# Supplementary figures and images for: BAG3 as a novel prognostic biomarker in kidney renal clear cell carcinoma correlating with immune infiltrates
Source: Eur J Med Res. 2024 Feb 1;29:93. doi: 10.1186/s40001-024-01687-w (PMC10832118; doi:10.1186/s40001-024-01687-w)

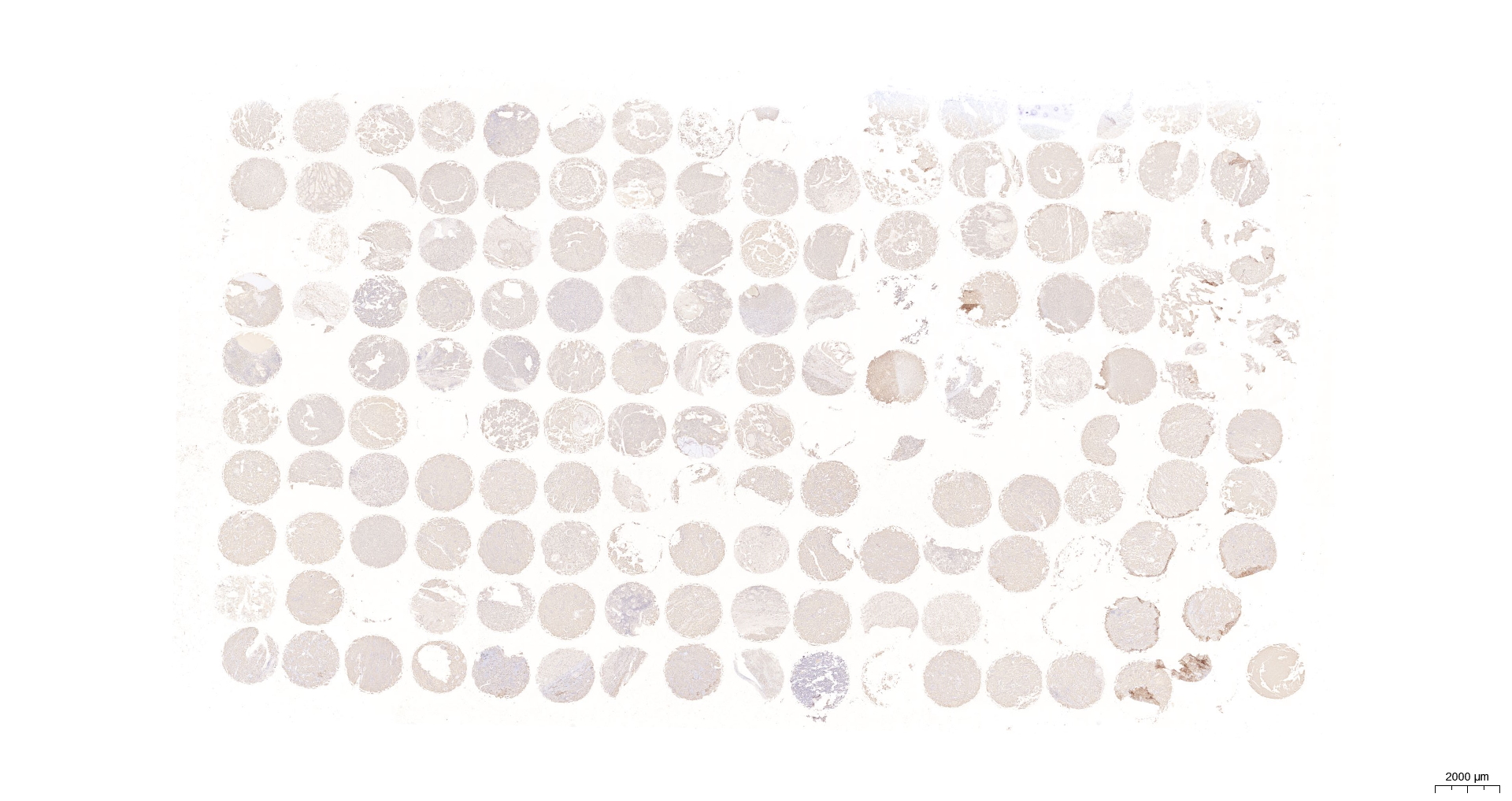

Supplement: Supplementary file 4 — Additional file 4. Fig S1. Tissue microarray. [file 40001_2024_1687_MOESM4_ESM.jpg]
